# Case Report: Selumetinib as a neoadjuvant treatment for the removal of a PN

**DOI:** 10.3389/fped.2026.1894268

**Published:** 2026-09-01

**Authors:** Ciavarelli Patricia, Mezmezian Mónica Beatriz, Daniel Yedlin, Paloma Amarillo

**Affiliations:** 1 Division of Neurosurgery and Multidisciplinary Center of Neurofibromatosis, School of Medicine, Hospital de Clínicas, Universidad de Buenos Aires, Buenos Aires, Argentina; 2 Laboratory of Neuropathology and Molecular Biology, Fleni, Buenos Aires, Argentina; 3 Neuro-orthopedic Department, Fleni, Buenos Aires, Argentina; 4 Department of Pediatric Oncology, Hospital Pereira Rossell, Fundación Pérez Scremini, Montevideo, Uruguay

**Keywords:** neoadjuvant treatment, neurofibromatosis type 1, plexiform neurofibroma, selumetinib, surgery

## Abstract

Neurofibromatosis type 1 (NF1) is an autosomal dominant genetic disorder that presents with benign tumors of the peripheral nerve sheath, named plexiform neurofibromas. Progressive tumor growth may cause pain, physical disfigurement, and compression of adjacent structures, potentially impairing normal development during childhood. We report the case of a 13-year-old patient with a plexiform neurofibroma in the right foot, which had been growing since the first months of life and compromised the talocalcaneal joint, severely impairing the patient's quality of life. The tumor was initially deemed inoperable due to the high risk of bleeding and impairment of the functional structure. Therefore, treatment with selumetinib was started. After nearly a year, the tumor volume had decreased significantly, allowing its resection and orthopedic reconstruction surgery. Selumetinib was discontinued 3 years post-surgery to evaluate tumor stability; however, tumor regrowth occurred, and the treatment was resumed. This case highlights the neoadjuvant role of selumetinib in transforming a tumor from inoperable to operable, resulting in a substantial improvement in the patient's quality of life.

## Introduction

Neurofibromatosis type 1 (NF1) is an autosomal dominant genetic disorder caused by pathogenic variants in the tumor suppressor *NF1* gene, which encodes a large protein, named neurofibromin, involved in the specific inhibition of RAS. NF1 deficiency leads to persistent RAS activation, causing overactivation of oncogenic pathways, such as the rapidly accelerated fibrosarcoma (RAF)/mitogen-actived protein kinase kinase (MEK)/extracellular signal-regulated kinase (ERK) pathway, which promotes cellular proliferation (1).

The clinical expression of NF1 includes hyperpigmentary abnormalities of the skin (café-au-lait macules and inguinal/axillary freckling), iris hamartomas, optic nerve glioma, choroidal abnormalities, bone disorders, and the growth of benign peripheral nerve sheath tumors (neurofibromas), which are classified into several tumor subtypes, including plexiform neurofibromas (PNs). This type of tumor can cause pain, compression of adjacent structures, and physical disfigurement, severely affecting the quality of life (QoL) of patients, including leading to psychological disorders (2, 3). In addition, tumor growth during childhood can affect the shape and alignment of growing bone, which is sensitive to surrounding mechanical environment forces (4).

Until recently, the only treatment for patients with NF1 and plexiform neurofibromas was surgical resection of the tumors. However, surgical intervention carries several risks, and a significant proportion of plexiform neurofibromas are considered inoperable. Molecularly targeted therapies against the mitogen-activated protein kinase enzymes MEK1 and/or MEK2 using MEK inhibitors have been developed. Selumetinib is the first drug approved by the Food and Drug Administration, European Medicines Agency, and Administración Nacional de Medicamentos for the treatment of symptomatic and inoperable plexiform neurofibromas in pediatric patients with NF1 (5, 6).

In this clinical case report, we demonstrate the value of selumetinib therapy in reducing tumor volume, allowing for plexiform neurofibroma resection and orthopedic reconstructive surgery that were previously considered infeasible.

## Clinical report presentation

At 3 months of age, the patient's mother perceived a difference in the infant’s ankles. The parents could not distinguish the right foot arch and the infant’s right foot had a rounded appearance. In addition, the mother noticed small bumps under the skin, similar to insect bites but without the reddish color. Moreover, the patient had café-au-lait spots on her skin. At 6 months of age, she was diagnosed with NF1. At the age of 3 years, a foot tumor biopsy confirmed suspicion of a plexiform neurofibroma. This tumor, located in her ankle, has grown since birth, leading to increasing deformity.

At 5 years old, the patient underwent Achilles tendon lengthening surgery due to hindfoot equinus deformity.

At 11 years old, the patient presented with an increase in the volume of the plexiform neurofibroma, which affected her gait and resulted in a convex foot with hindfoot valgus, forefoot abduction, and limited ankle dorsiflexion, leading to difficulty wearing footwear.

**Figure 1:**
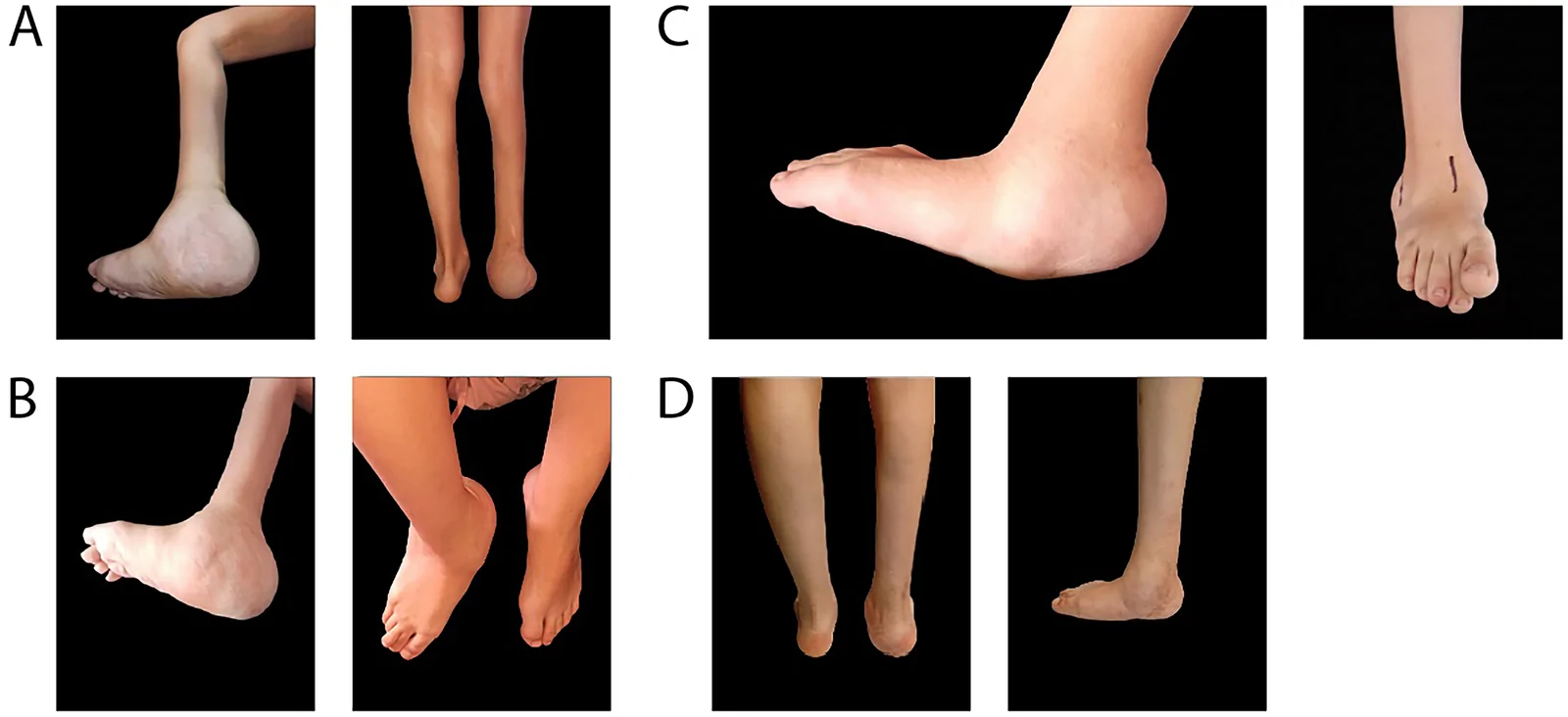
Images of the effects of the pharmacological and surgical treatments at the following timepoints: at the initiation of selumetinib therapy **(A)**, after 9 months of therapy **(B)**, after 12 months of therapy before surgery **(C)**, and after 40 months of selumetinib therapy and 28 months post-surgery **(D)**.

At the age of 13 years, magnetic resonance (MR) angiography of the lower limb showed extensive vascularization of the plexiform neurofibroma (images not available). MRI demonstrated that the tumor was located within the subcutaneous tissue of the posteromedial foot, with extension into the posterior compartment of the distal leg (involving the soleus, flexor muscles, tibialis posterior, and peroneal muscles) and the plantar musculature of the foot. The lesion measured approximately 145 mm × 94 mm × 65 mm (longitudinal (L) × transversal (T) × anteroposterior (AP)). Given the extent to which the tumor encased the functional structures of the ankle joint and its extensive vascularization, it was deemed inoperable by the multidisciplinary surgical team due to the high risk of irreversibly damaging critical nerves required for foot function during reconstructive surgery. In addition, to rule out potential tumor malignancy, a [18F] fluorodeoxyglucose-positron emission tomography (PET) scan-CT scan reported negative results [maximum standardized uptake value (SUVmax) of 2.9], with the tumor region found to be mildly hypermetabolic. This clinical scenario was deemed suitable for treatment with selumetinib (Figure 1A).

Regarding the patient's QoL, she did not experience pain in a rest position. However, when she was walking, she expressed persistent mild pain (3–4 on a 10-point Numeric Rating Scale). The foot deformity prevented her from wearing regular footwear, jogging, and participating in sports activities.

At the age of 14, oncologic treatment with selumetinib (25 mg/m^2^ twice daily) was initiated to reduce the volume of the tumor. Early treatment-related adverse effects included dry skin, an acneiform rash, and transient grade 1–2 vomiting and diarrhea, which did not require treatment modification. Comprehensive cardiological, hematological, renal, ophthalmological, hepatic, and dermatological assessments were conducted, revealing no abnormalities in the evaluated indicators or biomarkers.

**Figure 2:**
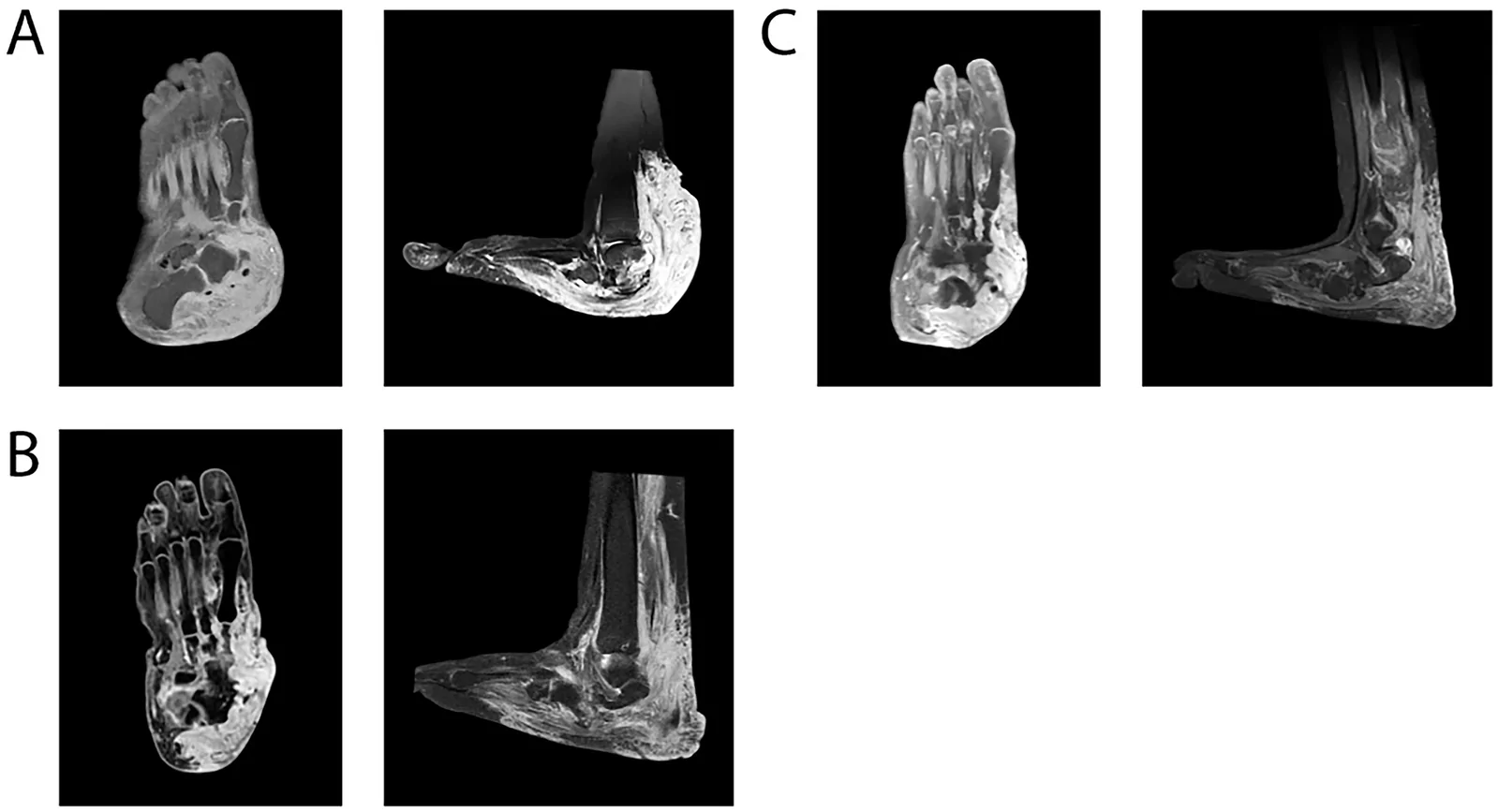
Magnetic resonance images in T1, T2, or diffusion mode after 9 months of selumetinib therapy **(A)**, after 12 months of selumetinib therapy before surgery **(B)**, and 40 months after selumetinib therapy and at 28 months post-surgery **(C)**.

After almost 1 year of treatment with selumetinib, we observed a significant reduction in the tumor volume (Figures 1B,C). At this time, a new magnetic resonance imaging scan revealed a solid mass consistent with a plexiform neurofibroma located in the subcutaneous tissue on the posteromedial side of the foot, involving the muscular structures of the posterior compartment of the distal third of the leg and the plantar region of the foot. Moreover, the scan showed morphological alterations of the tarsal bones, with dislocation of the talocalcaneal joint and subluxation of the talonavicular joint (Figure 2A).

Prior to surgery, the tumor exhibited a markedly softer consistency on palpation compared with the baseline before selumetinib treatment.

**Figure 3:**
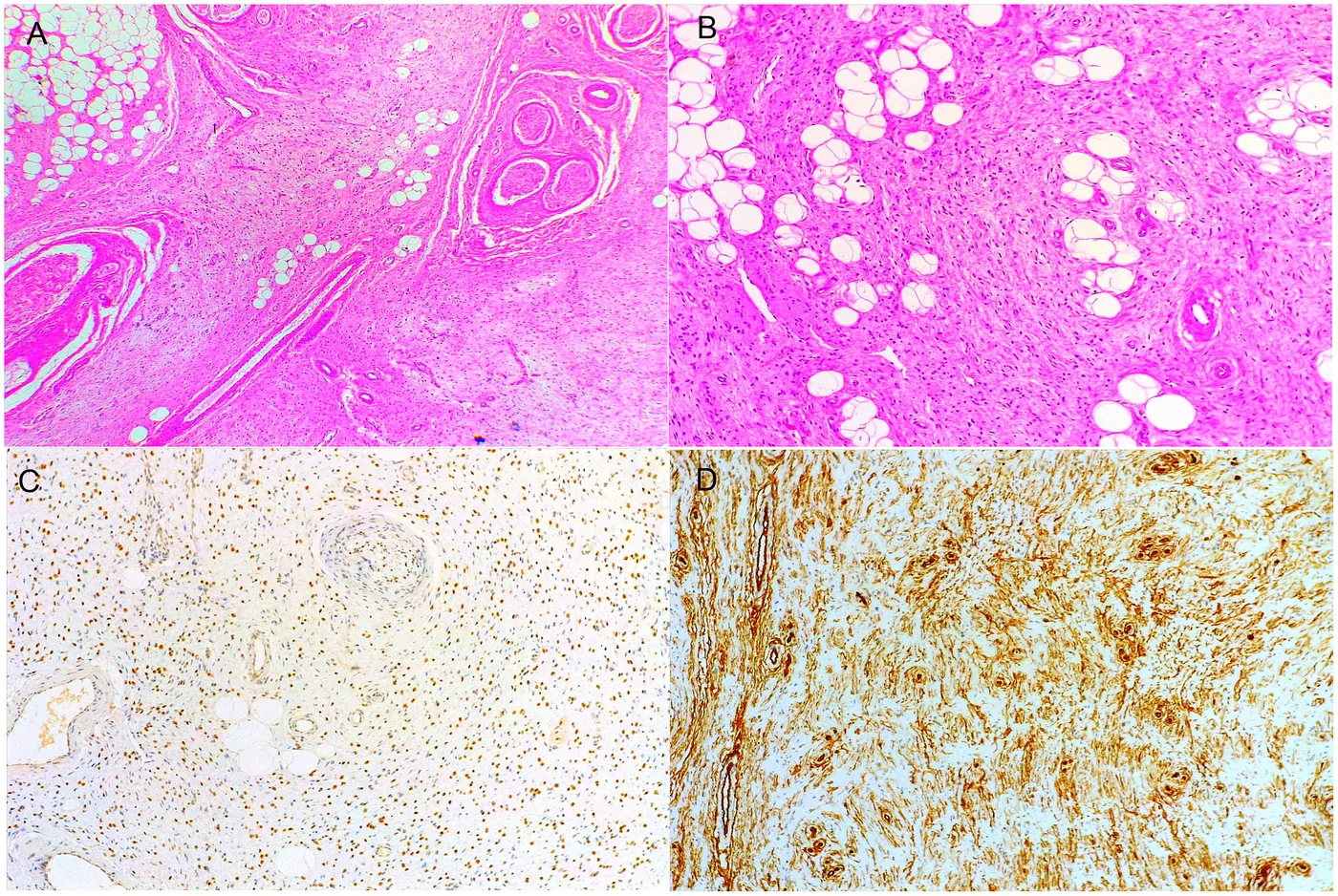
Representative histopathological images of a biopsy specimen from a neurofibroma obtained during surgical intervention. (**A**, **B)** H&E staining shows diffuse neoplastic proliferation of moderate cellularity composed of cells with ovoid to comma-shaped nuclei, moderate pleomorphism, and short spindle-shaped eosinophilic cytoplasm. The tumor infiltrates the fibroadipose tissue. Some enlarged nerve fibers are present. No mitotic figures, necrosis, or marked nuclear atypia are identified. (**C)** SOX10 shows numerous positive cells. (**D**) CD34 is positive in a lattice-like pattern. Objective magnification is 4× in **A** and 10× in **B**, **C**, and **D**.

The oncological surgery consisted of partial resection of the neurofibroma, primarily in the internal retromalleolar region of the foot. The orthopedic surgery consisted of lengthening the extensor tendons of the toes and ankle, resection of the navicular bone, lengthening of the Achilles tendon and common flexor tendons of the toes, calcaneocuboid arthrodesis, reorientation of the talus to a horizontal position, temporary fixation with Kirschner wires (K-wires), and a sliding calcaneal osteotomy using temporary fixation with K-wires (Figures 1D, 2B). The histopathological examination confirmed the tumor as a plexiform neurofibroma with diffuse and infiltrative extension (Figure 3). After surgery, the PN was estimated to be 138 mm × 66 mm × 54 mm (L × T × AP) in size. Selumetinib treatment was not interrupted due to surgery.

The patient's QoL improved substantially. A complete resolution of pain enabled her to wear normal shoes, resume physical exercise, and recover functional independence in daily activities.

**Figure 4:**
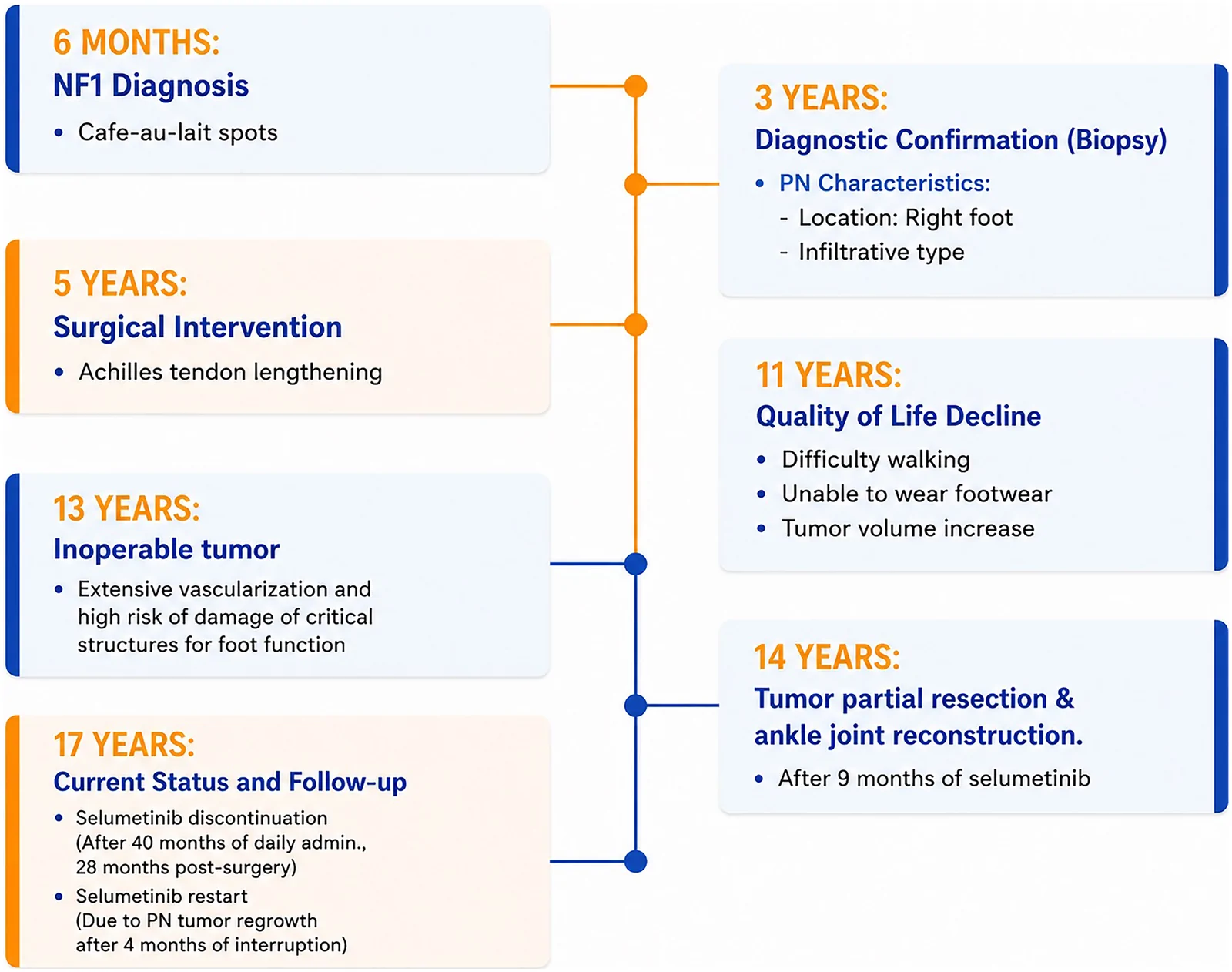
Milestones in the diagnosis and management of NF1 across the patient's lifespan.

Almost 4 years after beginning selumetinib treatment, the residual plexiform neurofibroma was stable in size and no associated toxicity was reported. Thus, it was decided to discontinue therapy with selumetinib (Figure 2C). However, 4 months after treatment interruption, an increase in plexiform neurofibroma volume was suspected and confirmed by MRI [148 mm × 90 mm × 50 mm (L × T × AP)]. Considering this result, selumetinib treatment was resumed (Figure 4).

## Discussion

This case report shows that the combined use of selumetinib and oncological-orthopedic reconstructive surgery as a therapeutic strategy led to a significant improvement in the patient’s quality of life, with the the patient even able to practice skating.

The clinical studies supporting the indication of selumetinib in pediatric patients with NF1 and inoperable plexiform neurofibromas established the initial paradigm for its use, positioning selumetinib as the first-line therapy when surgical intervention is not feasible (6, 7).

In this case report, selumetinib was used as a neoadjuvant therapy, contributing to creating a new paradigm and leading to a substantial reduction in tumor size, which reduced the risk of bleeding complications during surgery. Recently, Passos and colleagues published an observational study of 54 patients, evaluating the effect of combined use of selumetinib and tumor surgery on clinical outcomes. Surgery was performed during selumetinib treatment in only five patients, with a variable degree of response measured by changes in tumor volume (8). In our case, the neoadjuvant use of selumetinib and oncological surgery resulted in clear clinical benefits for the patient; tumor stabilization was not achieved due to the absence of an MEK inhibitor. Real-world evidence shows that the combined therapy of selumetinib and surgery plays a relevant role in the management of PNs in patients with NF1, providing better disease control and symptom improvement.

In this clinical case, we consistently observed that adverse events associated with prolonged selumetinib use (more than 4 years) had a mild impact on the patient’s quality of life, consistent with previously published observations (9).

We consider the use of selumetinib highly valuable for preventing excessive tumor growth and its consequences during childhood until the patient reaches an appropriate age for surgery (if it is considered an option). In fact, the benefits of selumetinib as a preventive agent against morbidities related to plexiform neurofibroma have been previously described in pediatric patients with NF1 and inoperable asymptomatic plexiform tumors, with a reduction in tumor size and no new symptoms appearing during selumetinib administration in 72% of cases (10).

## Conclusion

This case report showed that a therapeutic strategy combining selumetinib and reconstructive surgery resulted in a significant improvement in the patient's quality of life. We observed in this case that selumetinib may facilitate the transformation of an inoperable tumor into an operable one.

## Data Availability

The original contributions presented in the study are included in the article/Supplementary Material, further inquiries can be directed to the corresponding author.
